# Investigation of the Shrinkage and Air Permeability of Woolen Blankets and Blankets Made with Regenerated Wool

**DOI:** 10.3390/ma15103596

**Published:** 2022-05-18

**Authors:** Eglė Kumpikaitė, Ginta Laureckienė, Daiva Milašienė, Stasė Petraitienė

**Affiliations:** 1 Department of Production Engineering, Faculty of Mechanical Engineering and Design, Kaunas University of Technology, Studentų Str. 56, LT-51424 Kaunas, Lithuania; ginta.laureckiene@ktu.lt (G.L.); daiva.milasiene@ktu.lt (D.M.); 2 Department of Applied Mathematics, Faculty of Mathematics and Natural Sciences, Kaunas University of Technology, Studentų Str. 50, LT-51424 Kaunas, Lithuania; stase.petraitiene@ktu.lt

**Keywords:** woolen fabric, fabric with regenerated wool, blanket, shrinkage, air permeability

## Abstract

The aim of this article was to compare the shrinkage and air permeability properties of woolen fabrics and fabrics with regenerated wool woven with different weaves for establishing the suitability of regenerated wool for blankets. Two series of products with yarns of different raw materials were woven. One group of fabrics was woven with regenerated woolen yarn in the weft and woolen yarn in the warp. The other group of fabrics was woven only from 100% woolen yarns. The shrinkage in the directions of the warp and the weft and the air permeability of the fabrics with regenerated wool and 100% woolen fabrics with different weaves were investigated. The shrinkage in the directions of the warp and the weft in the fabrics with regenerated wool in the weft and 100% woolen fabrics depended on the float length in the weave. When the length of the weave increased, the shrinkage also increased. The air permeability value changed depending on the number of intersections and the float length. The fabrics with regenerated wool in the direction of the weft had higher air permeability. The Two-way analysis of variance (ANOVA) results showed that the weave influenced the shrinkage in the directions of the weft and warp, but the raw material had no influence on the shrinkage. The weave did not influence the air permeability, in contrast to the raw material. The shrinkage in the directions of the warp and weft and the air permeability did not depend on the interrelationships of the weave group and the raw material of the fabric.

## 1. Introduction

As the world develops and the population increases, our needs and use of food, clothes, and other products increase. The fashion and textile industry is one of the most polluting and resource-intensive fields across the world. Due to the popular “buy and throw” logic of high-speed fashion, textile production is increasing in capacity, posing a significant threat to our environment. These negative factors are encouraging the textile sector to become part of the circular economy, prolonging the life of clothing and other textiles, reusing fibers, and reducing the use of pure fibers. Although the secondary use of textile fibers requires significant work due to the fact that, in many cases, it is only possible to sort them by hand, more and more companies are gradually adopting this idea of a circular economy and trying to give new life to old products [1,2].

Unfortunately, investigations of regenerated woolen fibers are limited, mostly related to fiber origin, physical properties, and morphology [3,4,5]. However, investigations of the mechanical and comfort properties of regenerated woolen fibers as well as properties of woven fabrics from these fibers are lacking in the literature.

Shrinkage depends on the raw material, structure, and type of finishing of the woven fabric. One of the most important properties of fabrics during different finishing processes is fabric shrinkage in the directions of the warp and weft. For example, woolen fabrics extensively shrink during laundering [6,7,8,9], felting [8,10], tumble drying [8,11,12,13], whitening, biopolishing [8,14], and low-temperature dying with acid dyes [15]. Such laundering parameters as the liquor ratio, detergent concentration, pH, temperature, and time of treatment influence the shrinkage of woolen fabrics [16]. To make such fabrics shrink-resistant, different finishing can be customized [6,7,8,9,10,12,13,15,16,17,18,19,20,21,22].

Air permeability is one of the main thermal properties of fabrics, especially important for blankets. This property is highly related to the porosity of the fabric, of which the methods of establishment are described in reference [23]. The structural parameters, such as the raw material, linear density of the warp and weft yarns, number of yarn twists, warp and weft density, warp and weft crimp, and weave, influence the porosity and air permeability of fabrics [24,25,26,27,28,29,30,31,32,33,34]. The influence of the structure of woolen fabric on its comfort properties and shrinkage has been described in only a few works [25,26]; thus, such investigations are very important.

Many authors have analyzed the shrinkage and air permeability of woven woolen fabrics; however, a comparison of woolen fabrics to fabrics with regenerated wool has not been performed. However, this is particularly relevant when considering the replacement of woolen yarns with regenerated woolen yarns in order to increase the environmental friendliness of woolen fabrics. Moreover, the influence of the fabric structure on these two properties lacks investigation or has not been comprehensively researched. However, shrinkage is a very relevant property for all textile products because the final dimensions (length and width) of the product depend on shrinkage in the directions of the warp and weft. Thus, the important factor is not that shrinkage exists, but rather how much the measurements of the fabric change when different fabric structures and raw materials are used. Air permeability is very important for woolen blankets, and so changing woolen fibers into secondary used fibers would be very important to solve many ecological problems. Thus, the aim of this article was to compare the shrinkage and air permeability properties of woolen fabrics and fabrics with regenerated wool woven with different weaves in order to establish the suitability of regenerated wool for blankets.

## 2. Experiment

### 2.1. Object of the Investigation

Blankets were woven by the company Barker Textiles (Kaunas, Lithuania), which manufactures woolen, cotton, and synthetic blankets. Two series of products with different raw yarn materials were woven. Series 1 was woven with regenerated woolen yarn in the weft and woolen yarn in the warp. Series 2 was woven only from 100% woolen yarn. All of the parameters and the weaving loom, which were used for weaving the products of both series with different weaves, were the same, and only the raw material of the fabrics differed. The main parameters of the blankets are presented in Table 1.

The regenerated wool came from the Barker Textiles company’s pre-consumer waste. This waste is caused by weaving looms that use cut selvedges of fabric. The fabric that Barker Textiles mainly uses is 100% New Zealand wool, while the selvedge entanglement comprises PES yarn.

The company Manteco S.p.a. (Montemurlo, Italy) took the waste from Barker Textiles and mechanically shredded it with a converter machine. The wool waste goes between shafts with different garniture and speeds in the converter machine until the individual fibers are obtained. After this, because the waste from the company is not sorted by colors, it is dyed in black and mixed with raw white New Zealand wool. Then the fibers are spun into the yarns and after that into the woven fabrics. This process has been widely used in the Prato area since the 1800s.

**Figure 1:**
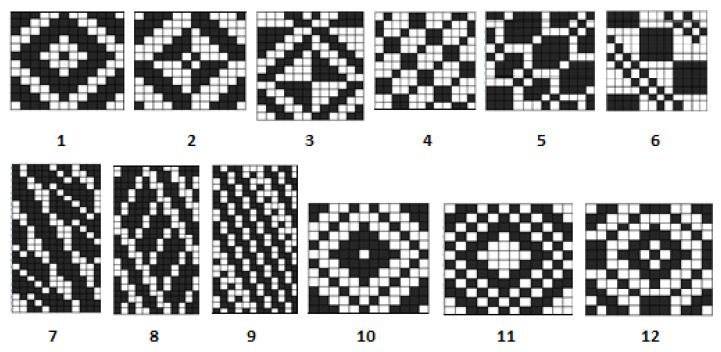
Weaves used for the experiment: 1, 2, 3—diamond twills; 4, 5, 6—derived baskets; 7, 8, 9—derived twills; 10, 11, 12—honeycombs.

**Figure 2:**
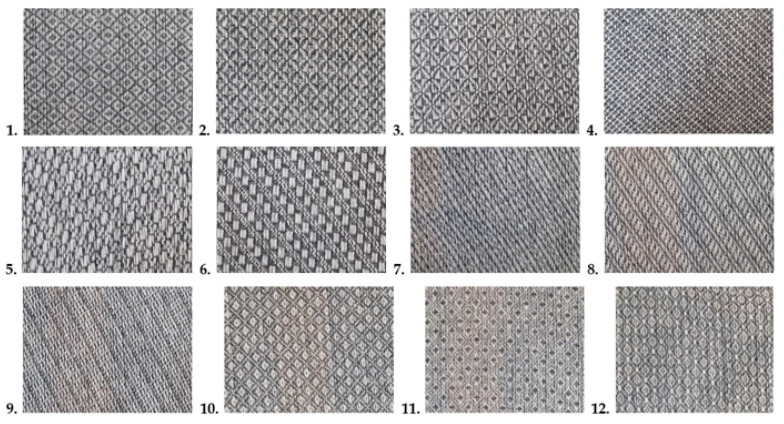
Pictures of the woven fabrics: 1, 2, 3—diamond twills; 4, 5, 6—derived baskets; 7, 8, 9—derived twills; 10, 11, 12—honeycombs.

Blankets of both series were woven with 12 different weaves, presented in Figure 1: three diamond twills, three derived baskets, three derived twills, and three honeycombs. Pictures of the woven fabrics with regenerated wool are presented in Figure 2. The pictures of woolen fabrics are not shown because colors of the warp and weft are the same (white) and the fabric pattern and texture of the fabrics would be not highlighted in the pictures.

Both series of woolen products were treated using the following finishing procedures: soaping 40 °C, fulling, washing 40 °C, softening 40 °C, wet raising, drying, and dry raising. All of these procedures were performed by the textile company Barker Textiles.

### 2.2. Weather Conditions of the Experiment

The samples were laid horizontally on the surface of a plane, and air could pass through the fabric. The samples were conditioned under standard weather conditions (Standard LST EN ISO 139: 2005/A1: 2011) before testing, i.e., the temperature was 20 ± 2 °C and the relative humidity was 65 ± 4%.

### 2.3. Method of Establishing Fabric Shrinkage

Evaluation of the changes in the dimensions after finishing the investigation, based on products made of natural or regenerated woolen fibers, was performed. The investigation of fabric shrinkage was performed according to Standard LST EN ISO 3759:2011. Preparation, marking, measurement before and after finishing, and finishing of the samples were performed for the investigation using the following tools:

- Metal ruler for measurement;
- Template with a marked location for the control points and a non-washable marker for marking;
- Equipment for finishing the woolen blankets.

The template used for marking had nine cut out points (diameter of the points was 3 mm). The bordering point was 50 mm from the edge, and the distance between the points was 100 mm. All marks and measurements were performed in the directions of the warp and weft.

The samples were kept under standard climatic conditions for the period of 4 h and were measured before the finishing processes.

Calculations of the measurement results of the samples in terms of the average shrinkage in the directions of the warp and the weft were made according to Standard LST EN ISO 3759:2011 and Equation (1):

(1) $$x=\frac{\left(x_{t}-x_{0}\right)}{x_{0}}100\%$$

where $x_{0}$ is the initial dimension and $x_{t}$ is the dimension measured after finishing.

Shrinkage was recorded as a percentage of the corresponding initial value. When the dimension decreased, it was marked with a minus sign (–), and when it increased (i.e., lengthened), it was marked with a plus sign (+).

### 2.4. Methods of Establishing Air Permeability

Seeking to establish the isolating properties of the blankets, an investigation of air permeability was performed according to Standard LST EN ISO 9237:1997. Debit of the air flow rate through a defined area of material at a specified pressure difference was measured.

The tests of air permeability were performed using an L14DR device (Karl Schroder KG, Weinheim, Germany). In this apparatus, the annular sample holders have a hole of 5, 20, 50, or 100 cm^2^. The pressure gauge connected to the test head has measuring scales of 50, 100, 200, or 500 Pa for determining the pressure difference with an accuracy of at least 2%.

A ring sample holder with a 5 cm^2^ hole was used for the test. The debit of air flow was measured under a pressure difference of 200 Pa. The tests were performed under standard climatic conditions, and the samples were kept under these conditions for a period of 24 h before the test.

A tensioned sample was placed in the ring sample holder, the air suction fan was switched on, and the air extraction was gradually increased until the selected pressure difference was reached under stable conditions. The test was performed 10 times for the same fabric in different places under the same conditions.

The air permeability *R*, dm^3^/(m^2^s), was calculated according to Equation (2):

(2) $$R=\frac{\bar{qv}}{A}167$$

where $\bar{qv}$ is the average of the air flow debit, dm^3^/min (L/min); *A* is the test area, cm^2^; and 167 is coefficient of conversion from dm^3^/cm^2^min or L/cm^2^min to dm^3^/(m^2^s).

## 3. Results and Discussion

One of the reasons of the differences in fabric properties of woolen and regenerated wool can be the different morphological and physical structure of the fibers. The thickness of the woolen fibers was approximately 34.43 mm, while that of the regenerated woolen fibers was 43.53 mm, but dispersion of the regenerated woolen fiber thickness was higher than that of the natural wool. The length of the natural woolen fibers was 47.00 mm, and the average length of the regenerated woolen fibers was 46.25 mm; however, the length of the regenerated wool varied in the range of 24.00–91.00 mm, i.e., the length of the fibers was very uneven. The reason is that the fiber of regenerated wool is damaged in the converter machine, some of fibers were broken, whereas the other fibers remained intact. Because of that, the physical parameters such as fiber thickness and length become uneven as discussed in the references [3,4,5].

**Figure 3:**
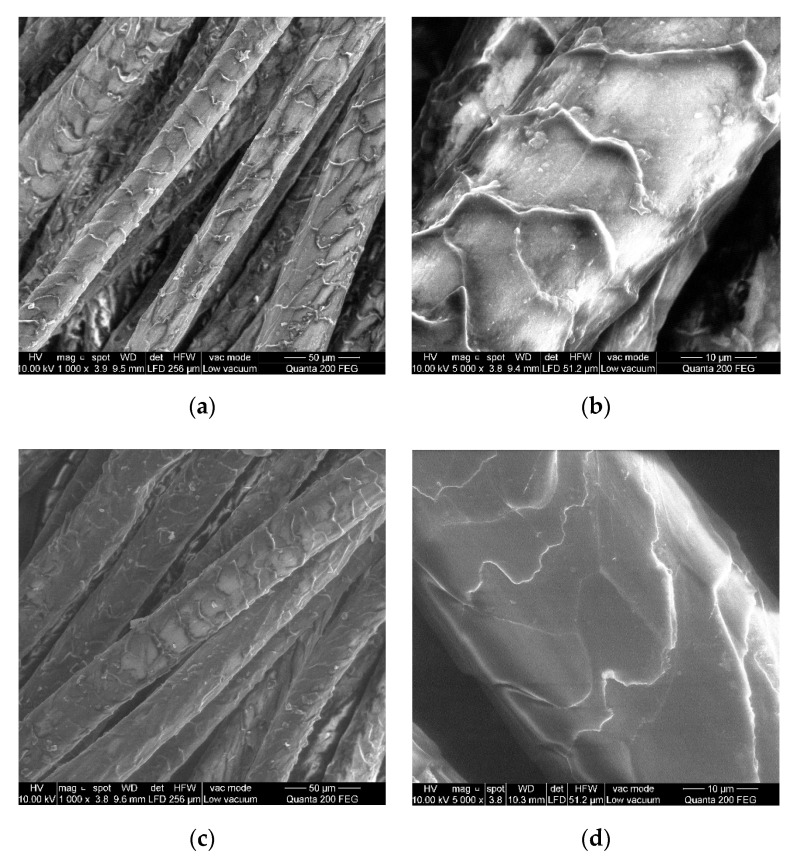
SEM images of (**a**) natural woolen fibers, magnification 1000×; (**b**) natural woolen fibers, magnification 5000×; (**c**) regenerated woolen fibers, magnification 1000×; (**d**) regenerated woolen fibers, magnification 5000×.

The differences between the natural and regenerated woolen fibers can also be seen in the SEM images in Figure 3. The flakes of the natural woolen fibers (Figure 3a,b) are brighter, more pronounced, and more embossed than the flakes of the regenerated wool (Figure 3c,d), which are duller and distributed closer to the fibers. This could be because the regenerated wool underwent significant mechanical and chemical effects during the converting process. As a result, the flakes would have rubbed off and stuck to the fibers, becoming less expressed than in the natural woolen fibers.

As mentioned above, fabric shrinkage in the directions of the warp and weft after finishing is very important because the final dimensions of a blanket depend on it. Therefore, it is very important to know the shrinkage in these directions after finishing before manufacturing the final product.

**Figure 4:**
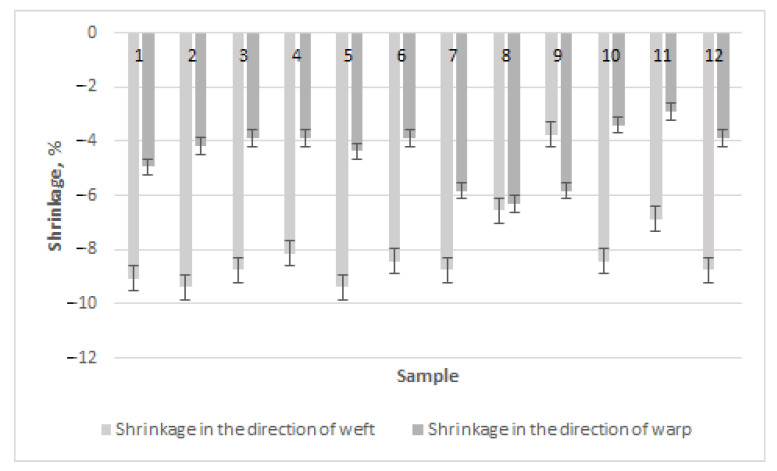
Shrinkage in the directions of the warp and the weft of Series 1.

Seeking to compare the results of the fabrics from regenerated and natural wool, column diagrams of shrinkage in the directions of the warp and the weft were drawn (Figure 4). The highest shrinkage in the direction of the weft occurred equally (–9.38%) for sample 2 (diamond twill weave) and sample 5 (derived basket weave) in Series 1 products with regenerated wool. Although the weave type of these two fabrics differed, floats of similar length (through three and four threads) predominated in the direction of the warp, leading to greater shrinkage in the direction of the weft. The lowest shrinkage in the direction of the weft in fabrics of Series 1 occurred for sample 9 with a derived twill weave (–3.75%). This weave had short floats in the directions of the warp and weft, i.e., floats through two and three threads predominated, and interlacing of one float in the weave existed. The highest shrinkage in the direction of the warp was obtained for sample 8 with a derived twill weave (–6.31%), which had especially long floats in the direction of the warp, while the lowest shrinkage occurred for sample 11 with a honeycomb weave (–2.91%), in which short floats and a high number of intersections in the directions of the warp and weft predominated. The authors investigated the shrinkage of fabrics woven in plain, different twill and basket weaves in the reference [16]. It was established that the shrinkage of woven fabric in both weft and warp directions depends on the length of weave floats. Woolen fabrics without surface treatment undergo major changes in their structure including the shrinkage after washing; thus, the different treatments can be used to reduce these undesirable properties [17,18,19,20]. Thus, the main result of our investigation is confirmation that the shrinkage of woolen fabric depends on fabric structure, especially on the fabric weave and the length of floats in the weave.

It can be seen from the diagram (Figure 4) that shrinkage in the direction of the weft was, in almost all cases, higher than in the direction of the warp—from 4% for weave 8 to 56% for weave 2. This could be because the weft threads were less tensioned than the warp threads and because the freer weft threads had the possibility of shrinking more than the tensioned ones. The ratio of shrinkage of weaves 1–6 and 10–12 was similar in the directions of the warp and the weft, i.e., approximately 50%. Shrinkage of weave 7 in the direction of the weft was also similar (–8.75%), but the ratio with warp shrinkage was lower than that of the earlier investigated weaves. This ratio of weave 8 was even lower—4%. However, the shrinkage tendencies of weave 9 in the directions of the warp and weft were opposite to those of other weaves, i.e., weft shrinkage was lower than warp shrinkage by 36%. These three weaves (7–9) were diagonals and their data differed from the data of the other weaves. This could be influenced by longer floats of one type in the direction of the warp and higher steps of the weave—the floats in the direction of the warp were longer than these in the direction of the weft, and because of this, shrinkage in the direction of the warp is easier. The authors of the references [10,16,18,19,20,22] investigated the shrinkage in weft and warp directions of different simple weaves such as plain, different twills, basket. They established that the weaves with longer floats in the directions of the warp and weft had the higher shrinkage than the fabrics with shorter floats. It was also established that in all cases, the shrinkage in the direction of the weft was higher than the shrinkage in the direction of the warp. These results are similar to those of our research, i.e., the shrinkage in both directions of the fabric depends on the length of the length of the weave float.

It can be seen from the diagram in Figure 4 that the highest shrinkage for Series 2 in the direction of the weft was obtained for sample 1, woven in a diamond twill weave (–10%). Therefore, the highest shrinkage in the direction of the weft occurred for the diamond twill weaves in the samples of Series 1. The lowest shrinkage in the direction of the weft was obtained for sample 9, as well as for those fabrics with a diagonal weave (–5.94%). The highest shrinkage in the direction of the warp was obtained for sample 8 with a diagonal weave (–6.8%), as well as for the fabrics of Series 1. The lowest shrinkage in the direction of the warp in the fabrics of Series 2 was obtained for samples 11 and 12 with a honeycomb weave with the same shrinkage (–3.11%). This result also corresponded to predomination of the warp shrinkage of Series 1 in honeycomb weaves, which had short floats and a high amount of interweaving. The influence of the weave of woolen fabrics on their shrinkage in the directions of the warp and weft has been investigated by other scientists [18,19,20]. It was estimated that the shrinkage increases when the float length of fabric weave decreases. The results of shrinkage can be compared to the results in [16], where the new method of shrinkage establishment with a launderometer for plain, twill 3/1, and basket 2/2 weaves fabrics was presented, i.e., 47% higher values than those of the usual method. Only the preliminary shrinkage results for regenerated wool fabrics were found in the references [1,2,3,4,5], but similar tendencies can be predicted for the regenerated wool fabrics as for the woolen fabrics.

**Figure 5:**
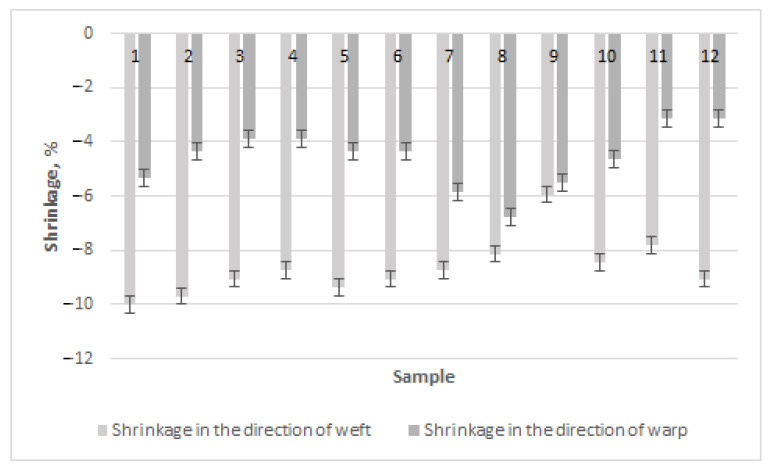
Shrinkage in the directions of the warp and weft of Series 2 fabrics.

When analyzing the shrinkage of Series 2 fabrics in the directions of the warp and weft (Figure 5), the same tendency as in the case of the fabrics of Series 1 was highlighted, i.e., in all cases, shrinkage in the direction of the weft was higher than that in the direction of the warp—from 8% for weave 9 to 61% for weave 12. As in the case of Series 1, weaves 7–9 differed as well—their ratio of shrinkage in the directions of the warp and weft was the lowest, from 8% for weave 9 to 34% for weave 7. Warp shrinkage of these weaves was higher than that of the other fabrics because their long floats were longer in the direction of the warp than in the direction of the weft. Therefore, the magnitude of shrinkage in both directions became similar. The ratio of shrinkage in the directions of the warp and weft of weaves 10–12 was the highest and ranged from 44% for weave 10 to 61% for weave 12. These were fabrics of honeycomb weave, whose float lengths were the same in the directions of the warp and weft. Because of these reasons, weft shrinkage was higher than warp shrinkage because the weft was less tensioned than the warp during weaving. The properties of regenerated wool fabrics were investigated in the references [2,3,4,5]. The results do not contradict our results. The tendencies of shrinkage in the direction of the weft being higher than in the direction of the warp were established in references [8,14]. The reason is that the weft was less tensioned in the fabric and it had a greater possibility of shrinking in the fabric than warp threads after applying the finishing processes. Thus, one of the findings of this investigation is that the fabric shrinks more in the direction of the weft than in the direction of the warp.

Weaves with longer floats had higher shrinkage, while lower shrinkage was established for weaves with shorter floats and a higher amount of interlacing. This defined better stability of the fabric dimensions. The same results were established for different weave types of Series 1 and 2. The highest shrinkage in the direction of the weft predominated in the weaves of diamond twill, while the fabrics of derived twill with short floats and high interlacing were the most stable. The results in the direction of the warp were the same—the highest shrinkage was obtained for diagonal weaves, and the lowest for honeycomb weaves. Comparing the shrinkage of the fabrics of Series 1 to those of Series 2, it can be seen that the fabrics of Series 1 with regenerated wool in the weft shrunk less than these of Series 2 (100% woolen). Thus, the main finding is that to avoid less shrinkage, it is recommended to use more regenerated wool in the fabric. Such results do not contradict earlier results of the references [10,16,18,19,20,22] for the shrinkage in the directions of the weft and warp of woolen fabrics and the results of the properties of regenerated woolen fabrics [1,2,3,4,5].

**Figure 6:**
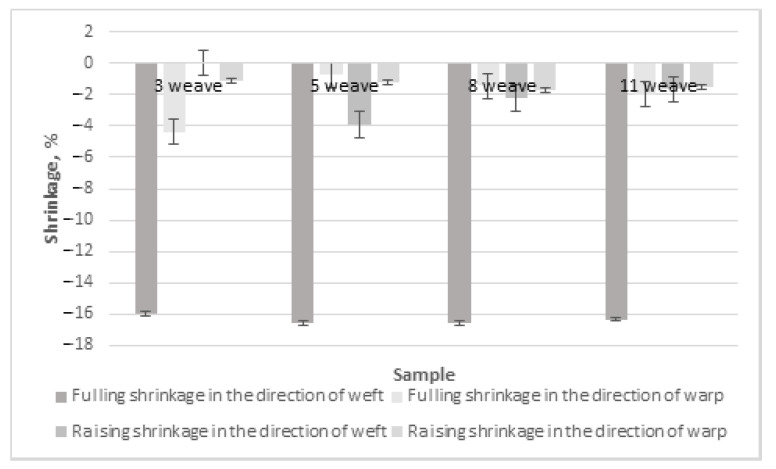
Fulling and raising shrinkage in the directions of the warp and weft of Series 1 fabrics with regenerated wool.

The finishing processes are also important for shrinkage in the directions of the warp and weft. Fulling and raising are the woolen fabric finishing processes that most influence fabric shrinkage. The results of fulling and raising shrinkage in the directions of the weft and warp for Series 1 fabrics with regenerated wool in the weft are presented in Figure 6. Only one fabric was investigated from each weave group because the earlier results showed similar across weave groups. It can be seen from Figure 6 that fulling shrinkage in the direction of the weft was the highest (from –16.00% for weave 3 to –16.56% for weaves 5 and 8) and the most expressed in comparison to shrinkage in the direction of the warp (from 0% for weave 3 to –3.91% for weave 5) and to raising shrinkage (from –0.74% for weave 5 to –4.38%) for weave 3 in the weft direction and from –1.13% for weave 3 to –1.76% for weave 8 in the warp direction). The results of the references [10,16] confirm that the woolen fabrics undergo the highest shrinkage during the fulling process because of wool fiber morphological structure, i.e., wool fiber is covered by scales, which caused fulling as well as shrinkage.

**Figure 7:**
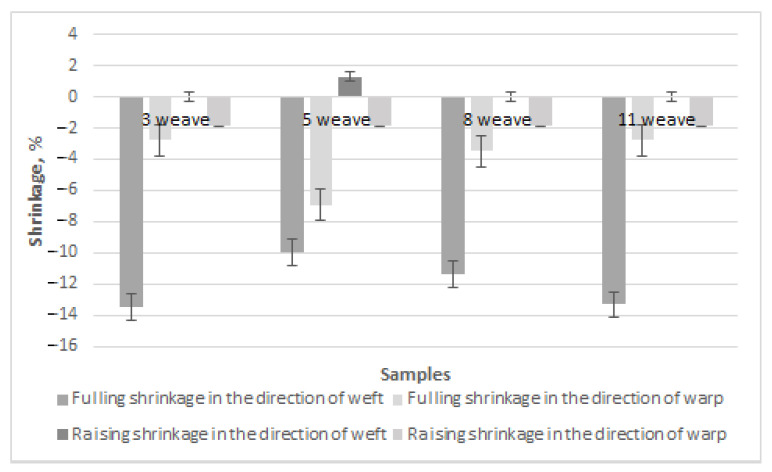
Fulling and raising shrinkage in the directions of the warp and weft of Series 2 100% woolen fabrics.

The fulling and raising shrinkage in the directions of the warp and weft of Series 2 100% woolen fabrics is presented in Figure 7. It can be seen that the fulling shrinkage in the direction of the weft also differed and was the highest (from –10.00% for weave 5 to –13.5% for weave 3). The fulling shrinkage in the direction of the warp is also expressed for weave 5 (–6.94%). This is similar to the results in the references [10,16].

When comparing the fulling and raising shrinkage in the directions of the warp and weft for fabrics of different raw materials, it can be seen that the fulling shrinkage in the direction of the weft was higher for fabrics with regenerated wool in the weft, from 15% for weave 3 to 39% for weave 5. This can be explained by the fibers of regenerated wool being shorter and injured during the recycling process. Because of this reason, short fibers of regenerated wool fulled and shrunk more. The results of the reference [16] are similar in that the fulling process had the highest influence on the shrinkage of woolen fabric. The authors of [10] suggest the use of laser treatment for plain weave woolen fabrics to avoid high fulling shrinkage instead of usual methods of shrinkage reducing. To make these fabrics shrink-resistant, different types of finishing, such as oxidative, enzymatic using different chemical means (keratinase and papain [17], protease [7], dicyandiamide, glycolic acid, alkali, hydrogen peroxide [8,14,15], Chlorine-Hercosett [12,13], laccase-assisted grafting of poly(tyrosine) [18], POSS^®^ nanomaterial [19], chitosan, wheat starch, and gum Arabic [9,20]), radiation, polymeric coatings, sol–gel coatings, plasma, and laser [10] treatments, softening, water-repellent finishing [21], and antimicrobial finishing [21,22], can be customized [6].

In order to determine whether fabric weave and raw material (independent variables) influence fabric shrinkage (dependent variable), two-way analysis of variance was performed. The hypotheses are presented in Table 2.

In order to check the hypotheses, ANOVA was performed. The results can be seen in Table 3.

The results showed that the hypothesis “There is no difference in the average yield for any group of weaves“ was not confirmed because *p_value_* = 0.012 < 0.05 (significance level); however, the alternate hypothesis “There is a difference in the average yield of the group of weaves“ was confirmed. This means that the fabric weave influenced the shrinkage in the direction of the weft. The hypothesis “There is no difference in the average yield of the raw material of the fabric” was accepted because *p_value_* = 0.1578 > 0.05. Thus, the raw material did not influence the shrinkage in the direction of the weft. The hypothesis “The effect of one independent variable on the average yield does not depend on the effect of the other independent variable” was confirmed because *p_value_* = 0.8352 > 0.05. In conclusion, the shrinkage in the weft direction did not depend on the interaction between the weave group and the raw material of the fabric.

**Figure 8:**
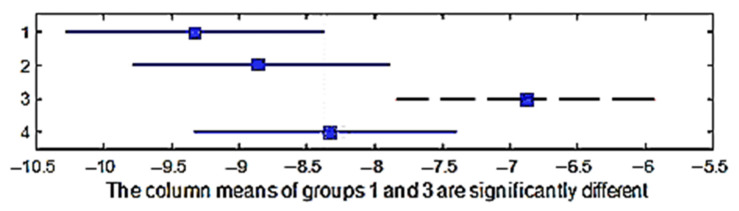
Confidence intervals of the shrinkage in the weft direction of different weave groups.

A multiple comparison test was performed in order to check the shrinkage in the direction of the weft between the four group of weaves. The *p*-values for the hypothesis test showed that the corresponding mean differences equal to zero were *p*_1–2_ = 0.8961, *p*_1–3_ = 0.0103, *p*_1–4_ = 0.3872, *p*_2–3_ = 0.0414, *p*_2–4_ = 0.7851, and *p*_3–4_ = 0.2214; the *p*_1–2_, *p*_1–4_, *p*_2–4_, *p*_3–4_ values were higher than 0.05 (significance level) and it can thus be stated that the averages of these groups were the same statistically, but the *p*_1–3_, *p*_2–3_ values were less than 0.05; thus, the average of the third group was significantly different. It means that the weave influenced the shrinkage in the direction of the weft. Figure 8 shows the multiple comparisons of the means.

**Figure 9:**
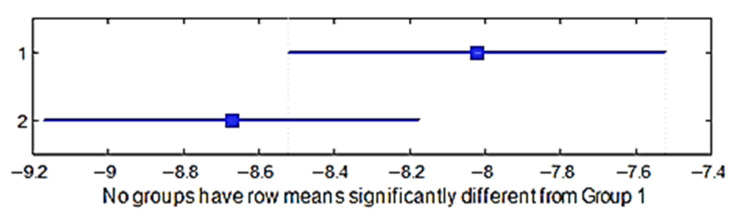
Confidence intervals of the shrinkage in the weft direction of different raw materials.

A multiple comparison test was performed to determine whether the shrinkage in the weft direction differed between the two raw materials of the fabric. The *p*-value = 0.1578 > 0.05; thus, the averages of these groups were statistically the same. This means that the raw material had no influence on the shrinkage in the weft direction (Figure 9).

The same hypotheses were proposed during the analysis of the shrinkage in the direction of the warp. The results of the ANOVA can be seen in Table 4.

The results showed that the hypothesis “There is no difference in the average yield for any group of weaves” was not accepted because *p*-value = 0 < 0.05. However, the alternate hypothesis “There is a difference in the average yield of group of weaves” was accepted. This means that the fabric weave influenced the shrinkage in the warp direction. The hypothesis “There is no difference in the average yield of the raw material of the fabric” was confirmed because *p*-value = 0.4906 > 0.05. Thus, the raw material did not influence the shrinkage in the direction of the warp. The hypothesis “The effect of one independent variable on the average yield does not depend on the effect of the other independent variable” was confirmed because *p*-value = 0.9947 > 0.05. Therefore, the shrinkage in the warp direction did not depend on the interaction between the weave group and raw material of the fabric.

**Figure 10:**
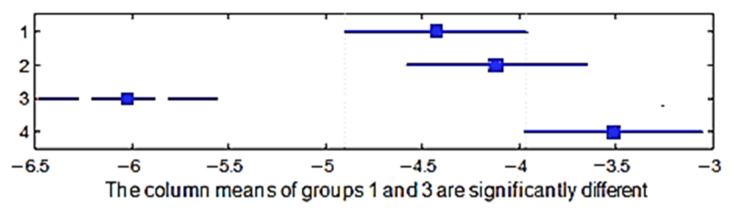
Confidence intervals of the shrinkage in the warp direction of different weave groups.

A multiple comparison test in order to determine the shrinkage in the direction of the warp between the four group of weaves was performed. The *p*-value for the hypothesis test showed that the corresponding mean difference was equal to zero; *p*_1–2_ = 0.7858, *p*_1–3_ = 0.0009, *p*_1–4_ = 0.0551, *p*_2–3_ = 0.0001, *p*_2–4_ = 0.2776, and *p*_3–4_ = 0.0000, and the *p*_1–2_, *p*_1–4_, *p*_2–4_ values were higher than 0.05 (significance level), which means that the averages of these weaves groups were the same statistically. However, the *p*_1–3_, *p*_2–3_, *p*_3–4_ values were lower than 0.05; therefore, the average of the third group was significantly different. This means that the weave influenced the shrinkage in the direction of the warp. Figure 10 shows the multiple comparisons of the means.

**Figure 11:**
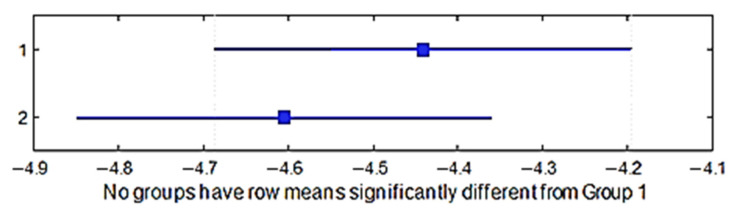
Confidence intervals of the shrinkage in the warp direction of different raw materials.

A multiple comparison test was performed in order to determine whether the shrinkage in the direction of the warp yield differed between two raw materials of the fabric. The *p*-value = 0.4906 > 0.05; thus, the averages of these groups did not differ statistically. This means that the raw material did not influence the shrinkage in the direction of the warp (Figure 11).

Thus, the ANOVA showed that the weave influenced the shrinkage in the directions of the weft and warp, but the raw material had no influence on the shrinkage. These results agree with the earlier results of our investigation that the shrinkage depended on the length of the floats in the fabric weave. The ANOVA also confirmed that the shrinkage did not depend on the raw material because the same tendencies of the shrinkage were obtained for both natural and regenerated wool fabrics. These results are the new findings of this research.

As mentioned above, the air permeability after finishing is very important because the thermal properties of the blanket depend on it. Therefore, it is very important to know the air permeability after finishing, before manufacturing the final product.

**Figure 12:**
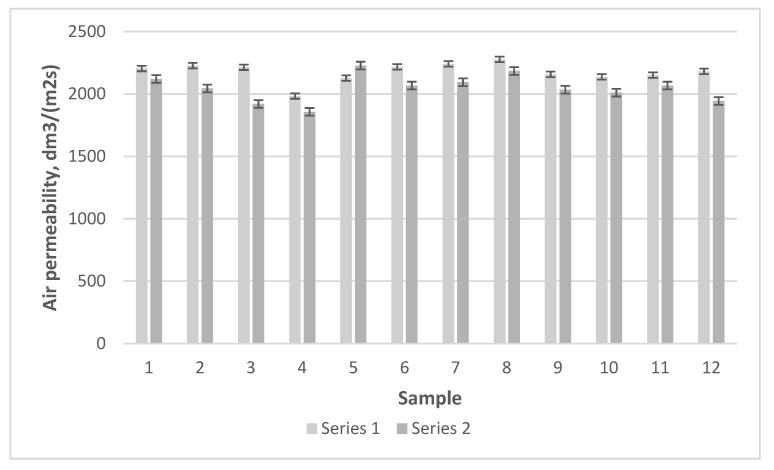
Air permeability of Series 1 and 2 fabrics.

To compare the results of regenerated and natural wool, a column diagram of air permeability according to weave type was drawn (Figure 12).

It can be seen from the presented results that the fabrics of Series 1 had higher air permeability—from 4% for weave 1 to 13% for weave 3. For regenerated wool, the wool scale may be destroyed during the preparation. As a result, entanglement should be more difficult for the adjacent woolen fibers or yarns; this may be one of the reasons for the better permeability. Therefore, the latter had higher air permeability than the woolen fabrics. Woolen yarns made from fibers of non-equal lengths had greater inequality and different cross-sections, and this resulted in higher air permeability, as can be seen in the case of the regenerated yarns. Based on a comparison of the fabrics according to weave type, sample 5 with a derived basket weave and sample 8 with a derived twill weave had the highest air permeability (2227.78 dm^3^/(m^2^ s) and 2184.36 dm^3^/(m^2^ s), respectively). This air permeability was lower by 2% than that of sample 5, but was higher than of the other samples. Sample 4 of a derived basket weave and sample 3 woven with a diamond twill weave had the lowest air permeability (1857.04 dm^3^/(m^2^ s) and 1920.50 dm^3^/(m^2^ s), respectively). The highest air permeability was established for samples 7 and 8 with derived twill weaves in Series 1 (2241.14 dm^3^/(m^2^ s) and 2277.88 dm^3^/(m^2^ s), respectively), while the lowest air permeability was found for sample 4 with a derived basket weave (1983.96 dm^3^/(m^2^ s)) and for sample 10 with a honeycomb weave (2137.60 dm^3^/(m^2^ s)). Thus, it can be concluded that the combined twill weave had the highest air permeability and the derived basket weave the lowest. These results correspond to the results in references [25,26], where the influence of different fabric structure parameters on fabric shrinkage in the directions of the warp and weft was investigated. The air permeability for plain and herringbone woolen fabrics was higher than that for other different twill weaves as presented in the reference [23].

The highest air permeability for the samples in Series 1 was obtained for sample 5 with a derived basket weave (2227.78 dm^3^/(m^2^ s)) because it is the weave in which long floats in the direction of the warp (through four threads) predominated; therefore, the air permeability was high. The air permeability can also be influenced by the warp floats being distributed in large elements through which air mainly passes. The air permeability was 2184.36 dm^3^/(m^2^s) in sample 8, woven with a combined twill weave, in which the number of floats in the direction of the warp predominated even for floats through six and four threads and in the direction of the weft through three and two threads; thus, the weave had long floats, especially in the direction of the warp, and this increased the air permeability. This weave had quite large weave elements with long floats, through which air could flow the easiest in the direction of the warp, as well as in the derived basket weave. The lowest air permeability was obtained for sample 4 of Series 1 with a derived basket weave (1857.04 dm^3^/(m^2^s)). However, this weave had smaller floats than sample 5. The weave also has a repeated repeat; thus, it is balanced very well and interlaces with one float in the warp that is often repeated. These aspects could have influenced the low air permeability. Low air permeability (1920.50 dm^3^/(m^2^s)) was also obtained for sample 3 woven with diamond twill. The given results of air permeability correspond to the studies published in references [23,24,25], where it was established that the value of air permeability changes depending on the increase in interlacing and the length of the floats. This was the reason for longer floats creating larger pores (spaces) in the weave and higher air permeability. One of the main findings of our investigation is that the air permeability of woolen fabrics depends on the length of the floats in the fabric weave.

The highest air permeability was obtained for samples 8 and 7 with natural woolen raw material from New Zealand of Series 2. Sample 8, woven with a combined twill weave, had an air permeability of 2277.88 dm^3^/(m^2^s), i.e., it was higher by 4% than that in Series 1. Sample 7, woven with a combined twill weave, had high air permeability (2241.14 dm^3^/(m^2^s)) due to its similar weave properties to sample 8. The weave also had floats through six threads in many places, and elements in the direction of the warp were distributed in large areas. This increased the air permeability. The lowest air permeability of Series 2 was obtained for samples 4 and 5 samples. Sample 4, similar to Series 1, had low air permeability (1983.96 dm^3^/(m^2^s)), while an air permeability of 2127.58 dm^3^/(m^2^s)) for sample 5 with a derived basket weave was obtained. The air permeability of woolen woven fabrics depends on their structure parameters, including the weave [24,25]. The given results correspond to previously described results, which state that weaves with longer floats have higher air permeability. Results in the reference [27] proved that the weave, weft yarn density, and finishing process influence the air permeability of woven fabrics. The 2/2 twill woven fabric, whose porosity was the lowest, had the lowest air permeability in comparison to other six combined weaves fabrics. These results confirm the dependence of fabric air permeability on the fabric weave.

The given results show that the results of air permeability have to be compared to the shrinkage in the direction of the warp (but not in the weft), i.e., the air permeability is higher when the shrinkage in the direction of the warp is lower. Thus, one of the main findings of our research is that the shrinkage in the direction of the warp had a greater influence than that in the weft direction when the air permeability is analyzed. In the reference [26], it was established that the raw material, linear density of the warp and weft yarn, number of yarn twists, warp and weft density, warp and weft crimp, as well as the weave influenced the porosity and air permeability of woolen woven fabrics of plain and twill weaves.

A comparison of fabrics woven from woolen yarn (Series 2) and regenerated wool yarn (Series 1) indicated that the latter had higher air permeability. For regenerated wool, the wool scale may be destroyed during the preparation. As a result, entanglement should be more difficult for the adjacent woolen fibers or yarns; this may be one of the reasons for the better permeability, and therefore the fabrics with regenerated wool had higher air permeability than the woolen fabrics. With the use of blends with other fibers [25,26,28,29] and surface treatment [30,31,33,34], the air permeability can be controlled. It was established that combined twill had the highest air permeability and derived basket weave the lowest. The air permeability was higher for those weaves in which the long floats in the direction of the warp predominated. The fact that the warp floats were distributed in large elements, through which the air mostly flows, could also have influenced the air permeability.

The same hypotheses were proposed when the air permeability was analyzed. ANOVA was performed (Table 5).

The results showed that the hypothesis “There is no difference in the average yield for any group of weaves” was accepted because *p*-value = 0.3942 > 0.05; thus, it can be stated that the weave did not influence the air permeability. The hypothesis “There is no difference in the average yield of the raw material of the fabric” was not confirmed because *p*-value = 0.0043 < 0.05. However, the alternate hypothesis “There is a difference in the average yield of the raw material of the fabric” was confirmed. Thus, the raw material influenced the air permeability. The hypothesis “The effect of one independent variable on the average yield does not depend on the effect of the other independent variable” was also accepted because *p*-value = 0.7003 > 0.05. Thus, it can be concluded that the air permeability did not depend on the interaction between the weave group and raw material of the fabric.

**Figure 13:**
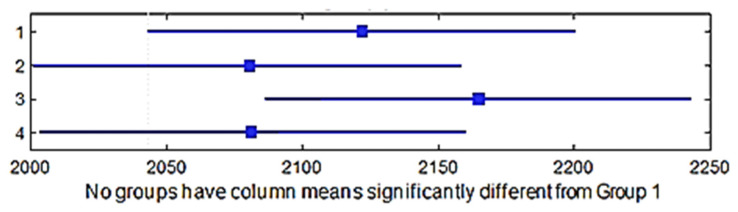
Confidence intervals of the air permeability of different weave groups.

A multiple comparison test was performed to establish whether the air permeability yield differed between the four groups of weaves. The *p*-value for the hypothesis test showed that the corresponding mean difference was equal to zero; *p*_1–2_ = 0.8716, *p*_1–3_ = 0.8628, *p*_1–4_ = 0.8843, *p*_2–3_ = 0.4396, *p*_2–4_ = 1.0000, and *p*_3–4_ = 0.4562. The *p*-values were higher than 0.05 (significance level); thus, there was no difference statistically, which indicates that the weaves of the fabric did not influence the air permeability. Figure 13 shows the multiple comparisons of the means.

**Figure 14:**
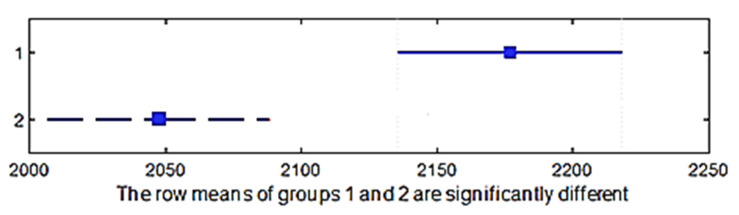
Confidence intervals of the air permeability of different raw materials.

A multiple comparison test was performed in order to see if the air permeability yield differed between the two raw materials of the fabric. The *p*-value = 0.0043 < 0.05; thus, the means were significantly different, which indicates that the air permeability yield differed across the two raw materials of the fabric (Figure 14).

The ANOVA showed that the weave did not influence the air permeability, but the raw material did.

The shrinkage in the directions of the warp and weft and the air permeability did not depend on the interrelationship of the weave group and the raw material of the fabric. Thus, it can be stated that searching for relationships between shrinkage and air permeability is not appropriate.

## 4. Conclusions

Upon investigation of shrinkage in the directions of the warp and weft, all fabrics were found to have shrunk after finishing. The reason is that the threads of the blankets relaxed during the finishing. Because of this, the threads crimped more and the fabric shrunk.

The shrinkage in the directions of the warp and weft in Series 1 and 2 depended on the used weave—more precisely, on the float length of the weave. It was determined that when the length of the float in the weave increased, the shrinkage also increased.

The investigation results showed that regenerated wool yarns should be used for better results in terms of shrinkage in the directions of the warp and weft.

It was found that when the warp floats in some used weaves were distributed in large elements, through which air flows easier, the air permeability was influenced.

After the tests of air permeability, it was established that the value of air permeability changed depending on the number of intersections and the float length.

The results of the shrinkage and air permeability of woolen fabrics and fabrics with regenerated wool in the weft indicated that regenerated wool can be used as a substitute for wool in blanket manufacturing because its properties differed from 4% to 13% for different weaves.

The statistical analysis showed that fabric shrinkage in the warp and weft directions depended on the fabric weave group, but the fabric weave group had no influence on the raw material used. Moreover, the raw materials of the fabrics investigated influenced the air permeability, but the air permeability did not depend on the weave group. In general, the ANOVA showed that the shrinkage in the weft and warp directions and air permeability did not depend on the interrelationship between the group of weaves and the raw material of the fabric.

## Figures and Tables

**Table 1:** Structural parameters of the products.

	Series 1	Series 2
**Raw material**	50% woolen yarn, 45% regenerated woolen yarn, and 5% other fibers	100% woolen yarn
**Linear density, tex**	166 × 2	166 × 2
**Color of yarn**	Grey	Grey
**Supplier**	Italy	New Zealand
**Warp setting, cm^–1^**	8.9	8.9
**Weft setting, cm^–1^**	6.5	6.5
**Weaving loom**	Dornier	Dornier
**Weft insertion**	Rapier	Rapier

**Table 2:** Hypotheses.

Null Hypothesis (H_0_)	Alternate Hypothesis (H_a_)
There is no difference in the average yield for any group of weaves.	There is a difference in the average yield for a group of weaves.
There is no difference in the average yield of the raw material of the fabric.	There is a difference in the average yield of the raw material of the fabric.
The effect of one independent variable on the average yield does not depend on the effect of the other independent variable.	There is an interaction effect between group of weaves and the raw material of the fabric on the average yield.

**Table 3:** ANOVA results for shrinkage in the weft direction.

Source	SS	df	MS	F	Prob > F
**Group of weaves**	20.2582	3	6.75273	5.04	0.012
**Raw material of the fabric**	2.94	1	2.94	2.2	0.1578
**Interaction**	1.1464	3	0.38213	0.29	0.8352
**Error**	21.4163	16	1.33852		
**Total**	45.7608	23			

**Table 4:** ANOVA results for shrinkage in the warp direction.

Source	SS	df	MS	F	Prob > F
**Group of weaves**	20.6132	3	6.87108	21.37	0
**Raw material of the fabric**	0.1601	1	0.16007	0.5	0.4906
**Interaction**	0.0233	3	0.00778	0.02	0.9947
**Error**	5.1438	16	0.32149		
**Total**	25.9404	23			

**Table 5:** ANOVA results for fabric air permeability.

Source	SS	df	MS	F	Prob > F
**Group of weaves**	28,842.8	3	9614.3	1.06	0.3942
**Raw material of the fabric**	100,073.2	1	100,073.2	11.02	0.0043
**Interaction**	13,100.4	3	4366.8	0.48	0.7003
**Error**	145,350	16	9084.4		
**Total**	287,366.4	23			

## Data Availability

All data are contained within this article.
